# Age and poverty status alter the coding and noncoding transcriptome

**DOI:** 10.18632/aging.101823

**Published:** 2019-02-17

**Authors:** Nicole Noren Hooten, Michele K. Evans

**Affiliations:** 1 Laboratory of Epidemiology and Population Science, National Institute on Aging, National Institutes of Health, Baltimore, MD 21224, USA

**Keywords:** lncRNA, ncRNA, noncoding RNA, aging, poverty, senescence

## Abstract

Emerging evidence indicates that noncoding RNAs play regulatory roles in aging and disease. The functional roles of long noncoding RNAs (lncRNAs) in physiology and disease are not completely understood. Little is known about lncRNAs in the context of human aging and socio-environmental conditions. Microarray profiling of lncRNAs and mRNAs from peripheral blood mononuclear cells from young and old white (n=16) and African American (AA) males (n=16) living above or below poverty from the Healthy Aging in Neighborhoods of Diversity across the Life Span study revealed changes in both lncRNAs and mRNAs with age and poverty status in white males, but not in AA males. We validated lncRNA changes in an expanded cohort (n=40); *CTD-3247F14.2, GAS5, H19, TERC* and *MEG3* changed significantly with age, whereas *AK022914,* *GAS5, KB-1047C11.2, MEG3* and *XLOC_003262* changed with poverty. Mitochondrial function and response to DNA damage and stress were pathways enriched in younger individuals. Response to stress, viral infection, and immune signals were pathways enriched in individuals living above poverty. These data show that both human age and a marker of social adversity influence lncRNA expression, which may provide insight about molecular pathways underlying aging and social factors that affect disparities in aging and disease.

## Introduction

Recent attention has focused on discovering new biomarkers that may serve as indicators of both health span and life span. DNA methylation, telomere erosion, DNA damage and repair, mitochondrial copy number and function are considered markers of biological aging [1]. However, it has been difficult to unequivocally identify a single universal biomarker to monitor aging differentiated from disease because there is considerable variation in the signs and symptoms of aging. Studies have also identified noncoding RNAs (ncRNAs), such as microRNAs (miRNAs), as relevant biomarkers of aging. We and others have shown that miRNA levels change during human aging [2–6]. Interestingly, these changes in miRNA levels can be modulated by interventions such as calorie restriction or metformin [7,8]. miRNAs are also key regulators of cellular processes important for aging such as cellular senescence [2]. Data from model systems also support a role for miRNAs in the aging process. For example, the *Caenorhabditis elegans* miRNA *lin-4* regulates lifespan through modulating expression of its target gene, *lin-14* [9]. Since this initial discovery several other miRNAs have been associated with regulating longevity in *C. elegans* [10].

Most recently, increasing interest in another class of ncRNAs, long ncRNAs (lncRNAs), has gained momentum as these molecules can also regulate gene expression at the transcriptional, post-transcriptional and translational levels [11,12]. Furthermore, lncRNAs have been linked to processes important for aging and age-related disease [13–15]. However, little is known about the global changes in lncRNA expression that occur with aging. Aging is a multi-factorial process marked by a fundamental decline in physiological responses and maintenance of tissue homeostasis and integrity. This decline of cell, tissue and organ function leads to an increase in a myriad of age-related diseases including cancer, type 2 diabetes mellitus, autoimmunity, infections, cardiovascular disease and ultimately mortality. Recent findings from model systems suggest that longevity and health span can be modulated by specific changes in gene expression patterns [10,16]. These patterns could be useful markers for identifying individuals in at-risk populations for the premature development of disease or monitoring adverse outcomes that may lead to early mortality.

Social determinants of health significantly influence the trajectory of aging, health status and outcomes among populations at risk for health disparities. It is well known that poverty and low socioeconomic status remain major risk factors for cardiovascular disease, chronic kidney disease and early mortality [17–19]. The weathering hypothesis suggests that socioeconomic disadvantage results in accelerated aging or premature declines in health status among African Americans (AAs) [20]. In the United States, AA men are particularly vulnerable to early mortality [21–24]. Therefore, it is important to identify the biological mechanisms and biologic risk factors through which social determinants of health accelerate aging phenotypes and trigger premature mortality. Adverse social conditions, including poverty, poor neighborhood conditions, discrimination, and crime are robust risk factors for negative health outcomes. Social adversity has been linked to gene expression changes in both children and adults [25–27]. Specifically, challenging social-environmental conditions initiate changes in gene expression among specific gene sets. The observed increase in mRNAs encoding inflammatory proteins and the decrease in mRNAs encoding immune-response proteins, termed the Conserved Transcriptional Response to Adversity (CTRA) [25–27], has been documented in several human and non-human models (for review [28]). These data hint that differences in gene expression may underlie racial and socioeconomic disparities in health.

Here we profiled both lncRNAs and mRNAs in the context of human aging, race and poverty. These lncRNAs may serve as potential biomarkers of susceptibility to age-related diseases. In addition, lncRNAs may be important promoters of the change in gene expression that results from exposure to various social-environmental conditions.

## RESULTS

### lncRNA changes with age and poverty

Although lncRNAs have been studied in the context of senescence and various other hallmarks of aging, little is known about whether lncRNA expression is altered with human age. Furthermore, since poverty is well known to influence accelerated development of age-associated chronic disease and premature mortality [29,30], we chose a sub-cohort of young and old AAs and whites from the Healthy Aging in Neighborhoods of Diversity across the Life Span (HANDLS) study that were both below and above poverty (Table 1). Poverty was designated as below poverty if the self-reported household income was below 125% of the 2004 Health and Human Services Poverty Guidelines at baseline recruitment. For our initial assessment of lncRNAs, we used the Arraystar human lncRNA microarray V3.0, which profiles about 30,586 lncRNAs and 26,109 coding transcripts. We selected young (30.6 yrs) and old males (63.3 years) that were either white or AA both below or above poverty (n=8/group Table 1).

**Table 1:** Demographic information for human cohorts.

**Microarray cohorts**		
**White males**	**Young**	**Old**
n	8	8
Age (mean(SD))	30.6 (0.5)	63.6 (0.5)
PovStat = Below (%)	3 (37.5)	4 (50)
		
**AA males**	**Young**	**Old**
n	8	8
Age (mean(SD))	30.6 (0.5)	63.5 (0.5)
PovStat = Below (%)	4 (50)	4 (50)
		
		
**Validation cohort**		
**White males**	**Young**	**Old**
n	20	20
Age (mean(SD))	32.1 (1.7)	62.6 (1.2)
PovStat = Below (%)	9 (45)	9 (45)

**Figure 1:**
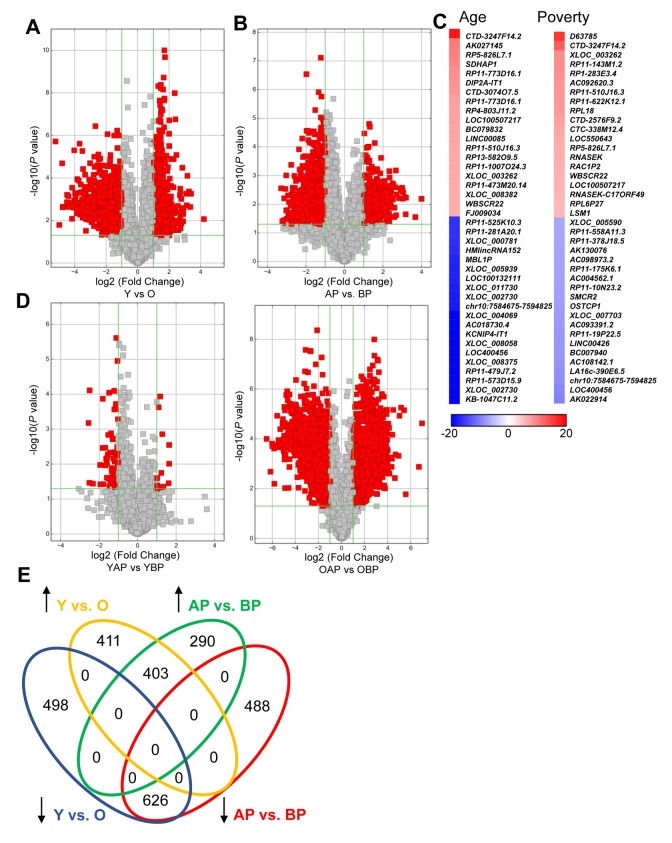
**Changes in lncRNA abundance with age and poverty.** The lncRNA expression levels in white young and old males (**A**) living above or below poverty (**B**) were assessed using microarrays (Table 1 for demographic details). Volcano plots show log2 fold change and *P* value for each lncRNA. Red indicates lncRNAs that were >2-fold change and *P*<0.05. (**A**,**B**,**D**) lncRNA comparisons between groups are indicated. (**C**) Heat maps indicate the top fold changed lncRNAs with age and poverty. These lncRNAs are also listed in Tables 2 and 3. (**E**) Venn diagram of the total number of significantly increased or decreased lncRNAs for each comparison. Y, young; O, old, AP, above poverty; BP, below poverty.

We found that a substantial number of lncRNAs were significantly altered with human age in white males (1938 lncRNAs; Figure 1A; Supplementary Table 1, Supplementary Table 2). In addition, poverty status also significantly changed the levels of lncRNAs in white males (1807 lncRNAs; Figure 1B; Supplementary Table 3, Supplementary Table 4). The top changes in lncRNA abundance with age and poverty are listed in Tables 2 and 3, respectively and shown in the heat maps in Figure 1C. A list of all significantly altered lncRNAs with age and poverty are found in Supplementary Table 1, Supplementary Table 2, Supplementary Table 3, Supplementary Table 4. Intriguingly, lncRNA changes with poverty were not observed in young individuals, but were robust among old individuals, consistent with the idea that poverty over the life span may greatly influence gene expression (Figure 1D). However, we did not assess childhood poverty or socioeconomic status. Comparison of the lncRNAs changed in abundance between poverty and age indicate that 1,029 lncRNAs that were significantly altered overlap with poverty and age (Figure 1E). We observed a different pattern for lncRNA differences with poverty and age in AA males (Supplementary Figure 1), where far fewer lncRNAs were significantly changed in abundance with age (only 26) and with poverty (245).

**Table 2:** Top changes in lncRNA abundance with age.

**Increased in Young**					**Decreased in Young**					
**Gene Symbol**	**Fold**	***P* value**	**Seqname**	**Source**	**Gene Symbol**	**Fold**	***P* value**	**Seqname**		**Source**
***CTD-3247F14.2***	18.50	0.008	ENST00000566457	GENCODE	***KB-1047C11.2***	37.34	0.000	ENST00000517655	GENCODE	
***AK027145***	10.52	0.001	uc002ywy.3	UCSC_knowngene	***XLOC_002730***	31.53	0.016	TCONS_00006917	LincRNAs; Cabili et al	
***RP5-826L7.1***	10.14	0.002	ENST00000446476	GENCODE	***RP11-573D15.9***	27.85	0.000	ENST00000577781	GENCODE	
***SDHAP1***	9.57	0.000	ENST00000440850	pseudogene	***RP11-479J7.2***	27.24	0.002	ENST00000425271	GENCODE	
***RP11-773D16.1***	8.94	0.000	ENST00000496359	GENCODE	***XLOC_008375***	26.51	0.002	TCONS_00018115	LincRNAs; Cabili et al	
***DIP2A-IT1***	8.67	0.002	NR_046400	RefSeq	***LOC400456***	22.55	0.001	NR_034095	RefSeq	
***CTD-3074O7.5***	8.47	0.001	ENST00000533502	GENCODE	***XLOC_008058***	22.39	0.001	TCONS_00017457	LincRNAs; Cabili et al	
***RP11-773D16.1***	7.60	0.003	ENST00000488805	GENCODE	***KCNIP4-IT1***	21.32	0.002	NR_002813	RefSeq	
***RP4-803J11.2***	7.46	0.005	ENST00000418348	GENCODE	***AC018730.4***	21.06	0.001	ENST00000454183	GENCODE	
***LOC100507217***	7.39	0.011	NR_037600	RefSeq	***XLOC_004069***	20.96	0.001	TCONS_00008575	LincRNAs; Cabili et al	
***BC079832***	7.16	0.000	uc003nhj.3	UCSC_knowngene	***chr10:7584675-7594825***	18.28	0.002	chr10:7584675-7594825-	LincRNAs; Cabili et al	
***LINC00085***	7.10	0.003	ENST00000573896	GENCODE	***XLOC_002730***	18.03	0.008	TCONS_00006916	LincRNAs; Cabili et al	
***RP11-510J16.3***	6.89	0.013	ENST00000564138	GENCODE	***XLOC_011730***	17.92	0.000	TCONS_00024847	LincRNAs; Cabili et al	
***RP13-582O9.5***	6.83	0.001	ENST00000521207	GENCODE	***LOC100132111***	17.58	0.002	NR_024237	RefSeq	
***RP11-1007O24.3***	6.64	0.005	ENST00000565181	GENCODE	***XLOC_005939***	17.49	0.002	TCONS_00011248	LincRNAs; Cabili et al	
***XLOC_003262***	6.59	0.023	TCONS_00006666	LincRNAs; Cabili et al	***MBL1P***	17.35	0.002	ENST00000480805	GENCODE	
***RP11-473M20.14***	6.55	0.000	ENST00000575139	GENCODE	***HMlincRNA152***	16.72	0.002	HMlincRNA152-	LincRNAs; Cabili et al	
***XLOC_008382***	6.53	0.000	TCONS_00018121	LincRNAs;Cabili et al	***XLOC_000781***	16.36	0.000	TCONS_00001451	LincRNAs; Cabili et al	
***WBSCR22***	6.52	0.008	NR_037776	RefSeq	***RP11-281A20.1***	16.34	0.002	ENST00000449730	GENCODE	
***FJ009034***	6.41	0.001	uc010wyg.2	UCSC_knowngene	***RP11-525K10.3***	16.31	0.001	ENST00000568776	GENCODE	

Top lncRNAs differentially expressed with age and *P*<0.05, Fold change >2 and FDR <0.1.

**Table 3:** Top changes in lncRNA abundance with poverty.

**Increased in Above Poverty**						**Decreased in Above Poverty**				
**Gene Symbol**	**Fold**	***P* value**	**Seqname**		**Source**	**Gene Symbol**	**Fold**	***P* value**	**Seqname**	**Source**
***D63785***	15.93	0.006	uc002hlt.1	UCSC_knowngene		***AK022914***	9.82	0.003	uc001vvr.1	UCSC_knowngene
***CTD-3247F14.2***	12.74	0.028	ENST00000566457	GENCODE		***LOC400456***	9.41	0.039	NR_034095	RefSeq
***XLOC_003262***	9.13	0.006	TCONS_00006666	LincRNAs; Cabili et al		***chr10:7584675-7594825***	8.88	0.036	chr10:7584675-7594825-	LincRNAs; Cabili et al
***RP11-143M1.2***	9.02	0.022	ENST00000442069	GENCODE		***LA16c-390E6.5***	8.81	0.006	ENST00000566287	GENCODE
***RP1-283E3.4***	8.86	0.006	ENST00000577672	GENCODE		***AC108142.1***	8.39	0.048	ENST00000507869	GENCODE
***AC092620.3***	8.23	0.004	ENST00000453636	GENCODE		***BC007940***	8.22	0.038	uc002xzq.1	UCSC_knowngene
***RP11-510J16.3***	8.15	0.006	ENST00000564138	GENCODE		***LINC00426***	8.17	0.001	ENST00000447147	GENCODE
***RP11-622K12.1***	8.05	0.003	ENST00000423617	GENCODE		***RP11-19P22.5***	8.14	0.017	ENST00000583179	GENCODE
***RPL18***	7.91	0.003	NR_073022	RefSeq		***AC093391.2***	8.01	0.016	ENST00000446492	GENCODE
***CTD-2576F9.2***	7.55	0.003	ENST00000568835	GENCODE		***XLOC_007703***	8.01	0.032	TCONS_00016773	LincRNAs; Cabili et al
***CTC-338M12.4***	7.04	0.006	ENST00000506340	GENCODE		***OSTCP1***	7.70	0.005	ENST00000522287	GENCODE
***LOC550643***	6.54	0.005	NR_015367	RefSeq		***SMCR2***	7.56	0.025	ENST00000456090	GENCODE
***RP5-826L7.1***	6.39	0.024	ENST00000446476	GENCODE		***RP11-10N23.2***	7.38	0.008	ENST00000520562	GENCODE
***RNASEK***	6.35	0.001	NR_037715	RefSeq		***AC004562.1***	7.27	0.024	ENST00000420427	GENCODE
***RAC1P2***	6.26	0.003	ENST00000511164	pseudogene		***RP11-175K6.1***	7.26	0.027	ENST00000523301	GENCODE
***WBSCR22***	6.01	0.013	NR_037776	RefSeq		***AC098973.2***	7.21	0.019	ENST00000414382	GENCODE
***LOC100507217***	6.00	0.028	NR_037600	RefSeq		***AK130076***	7.17	0.039	uc001kfc.1	UCSC_knowngene
***RNASEK-C17ORF49***	5.81	0.002	NR_037717	RefSeq		***RP11-378J18.5***	6.96	0.011	ENST00000433391	GENCODE
***RPL6P27***	5.66	0.007	ENST00000583065	GENCODE		***RP11-558A11.3***	6.91	0.040	ENST00000568458	GENCODE
***LSM1***	5.65	0.002	NR_045493	RefSeq		**XLOC_005590**	6.898	0.035	TCONS_00012060	LincRNAs; Cabili et al

Top lncRNAs differentially expressed with poverty and *P*<0.05, Fold change >2 and FDR <0.1.

**Figure 2:**
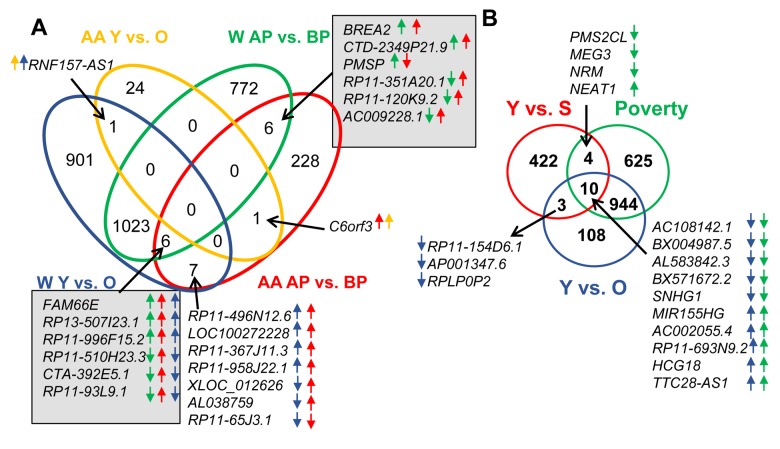
**Comparison of lncRNA level changes with age, poverty and senescence.** (**A**) Significantly changed lncRNA abundance was compared between African American (AA) males living below poverty (BP) and above poverty (AB) to white males (W) living below and above poverty. (**B**) Changes in lncRNAs levels in whites with poverty and age were also compared to significantly changed senescence-associated lncRNAs (SAL-RNAs) identified previously [31]. The comparisons in (**A**) were done using the Seqname and (**B**) using Gene symbol, hence the slight differences in numbers. Arrows indicate the direction of change (higher or lower) in the comparisons. The color of the arrow indicates the comparison examined.

To determine the effects of race on lncRNA expression, we compared lncRNAs that were altered by race. We found that 291 lncRNAs were altered between white and AA males. Furthermore, if we categorize based on race, poverty and age, we identified changes in lncRNA levels that occurred with both races comparing above and below poverty status. There were 12 lncRNAs that were altered with poverty in both races and these are indicated in gray boxes in Figure 2. Only one lncRNA, *RNF157-AS1*, changed significantly with age in both whites and AAs, and one lncRNA, *C6orf3,* changed significantly with both poverty and age in AAs (Figure 2A).

### Comparison to age-associated lncRNAs

Aging and poverty associated changes in lncRNA levels in whites were also compared to lncRNAs that have previously been described in the literature to be age-associated. Since senescent cells accumulate with age, and have been linked to a number of age-related pathologies, we first compared our dataset to lncRNAs that previously were found to be significantly changed in abundance during cellular senescence, termed senescence-associated lncRNAs (SAL-RNAs) [31]. Several poverty-associated lncRNAs (14) and aging-related lncRNAs (13) overlapped with the various SAL-RNAs (Figure 2B). There were 10 SAL-RNAs that overlapped with both comparisons (Figure 2B).

**Figure 3:**
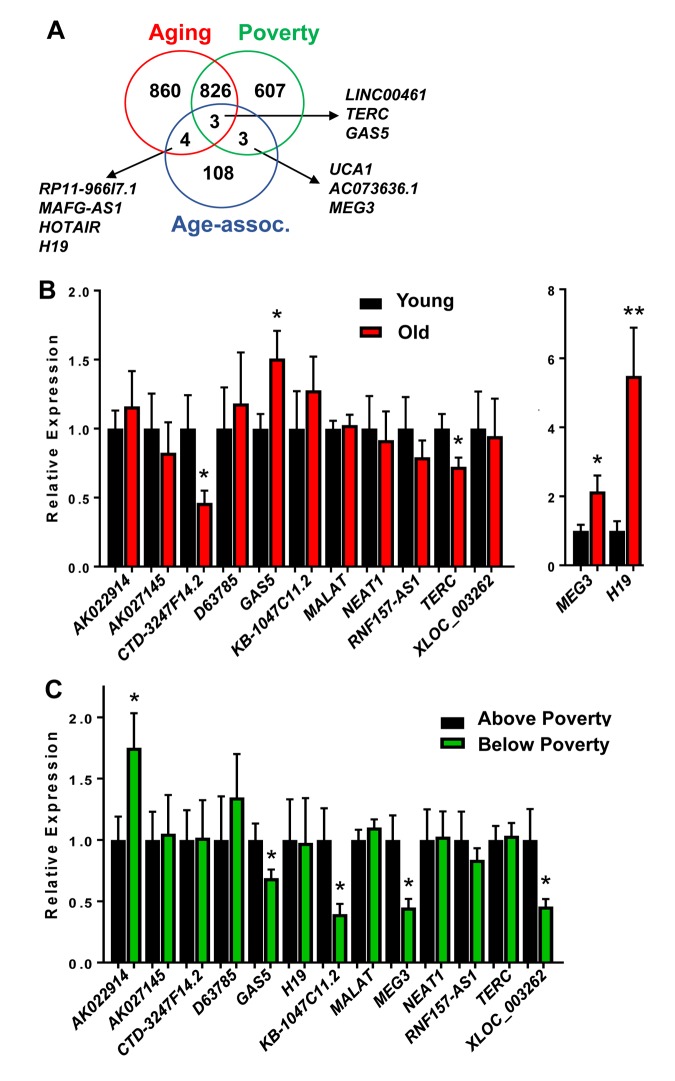
**Validation of age and poverty associated level changes in lncRNAs abundance.** (**A**) lncRNAs significantly changed in abundance with age and poverty in white males were compared to lncRNAs identified in the literature to be age-associated. Overlapping lncRNAs are shown. (**B**-**C**) RNA was isolated from PBMCs from young (mean=32.1 yrs) and old (mean=62.6 yrs) white males above and below poverty (n=40; see Table 1 for demographics). RNA was reverse transcribed and lncRNA levels were quantified by RT-qPCR.

We also further compared the lncRNAs we found to be significantly different to previously reported age-associated lncRNAs. These lncRNAs were compiled based on the regulation of various hallmarks of aging [13] or age-associated functions, including differences with aging in the achilles tendon [32], specific lncRNAs associated with regulating senescence [33,34], mouse premature aging and muscle wasting [35]. This list consisted of 118 “Age-associated lncRNAs” (Supplementary Table 5). Three lncRNAs were overlapping between these Age-associated lncRNAs and our poverty and aging lncRNAs and three were overlapping just with poverty and four just with age (Figure 3A).

### Validation of lncRNA changes with age and poverty

Several of these overlapping lncRNAs were chosen to further validate in an expanded cohort of individuals (n=40; Table 1). In addition, we also validated the top lncRNAs that were significantly changed in abundance with either age or poverty (Tables 2, 3). All significantly changed lncRNAs are listed in Supplementary Table 1, Supplementary Table 2, Supplementary Table 3, Supplementary Table 4. We designed primers for the top lncRNAs showing the most robust changes in levels and for various “Age-associated lncRNAs” including *H19, MEG3, TERC* and *GAS5*. We designed multiple primer sets for *XLOC-002730* and *LOC400456*, but we were unable to amplify these lncRNAs. We chose to further validate in the expanded cohort a total of 13 lncRNAs. *RNF157-AS1* was included since levels of this lncRNA were changed differentially with age in AAs and whites, but it was not significantly altered in this expanded cohort. Importantly, *CTD-3247F14.2 (*LNCipedia: *lnc-EGR3-1), GAS5, H19, TERC* and *MEG3* levels were all significantly changed with age (Figure 3B). *AK022914 (DUXAP9),* *GAS5, KB-1047C11.2 (*LNCipedia: *C8orf37-AS1:1), MEG3* and *XLOC_003262* (LNCipedia: *lnc-KY-1*) levels were significantly different with poverty (Figure 3C). Interestingly, *GAS5* binds and acts as a cytoplasmic decoy for the glucocorticoid receptor (GR) [36]. This observation is intriguing given that transcriptional changes in GR signaling pathways are part of the Conserved Transcriptional Response to Adversity (CTRA) in mice and humans [28].

### mRNA changes with age and poverty

**Figure 4:**
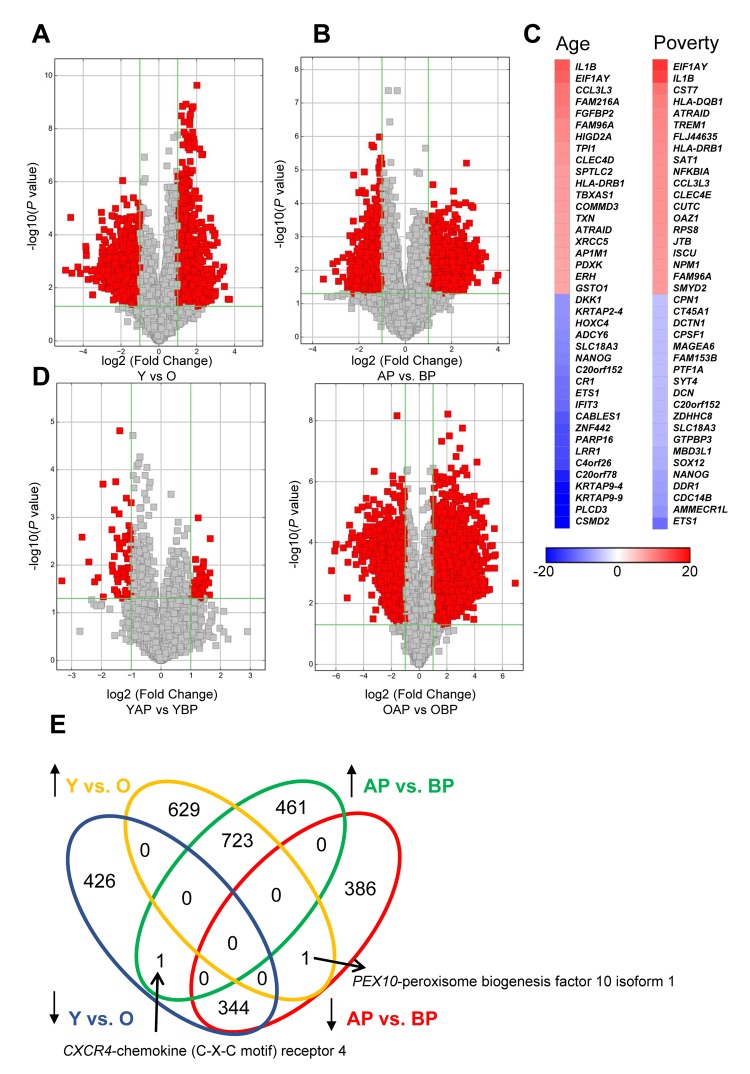
**Changes in mRNA expression levels with age and poverty.** The levels of expressed mRNAs in white young and old males (**A**) living above or below poverty (**B**) were assessed using microarrays. Volcano plots show log2 fold change and *P* value for each mRNA. Red indicates mRNAs that changed were >2-fold, with *P*<0.05. (**A**,**B**,**D**) Comparisons of significantly different mRNAs between groups are indicated. (**C**) Heat maps indicate the top fold changed mRNAs with age and poverty. These mRNAs are also listed in Tables 4 and 5. (**E**) Venn diagram of the total number of significantly increased or decreased mRNAs for each comparison. Y, young; O, old, AP, above poverty; BP, below poverty

In addition to examining lncRNA levels, we also wanted to further understand differences in the levels of expressed mRNAs with poverty and age. To do this, we examined mRNA expression levels by microarray and found that, similar to the lncRNAs, there were many significantly different mRNAs with both age and poverty in white males (Figure 4A,B; Supplementary Table 6, Supplementary Table 7, Supplementary Table 8, Supplementary Table 9). The top changes in mRNA abundance with age and poverty are listed in Tables 4 and 5, respectively and shown in the heat maps in Figure 4C. A list of all significantly altered mRNAs with age and poverty are found in Supplementary Table 6, Supplementary Table 7, Supplementary Table 8, Supplementary Table 9. Changes in expressed mRNAs with poverty were also more robust in the older individuals compared to the younger individuals, similar to our results with lncRNAs (Figure 4D). Many of the differentially abundant mRNAs were overlapping between poverty and age (Figure 4E). Interestingly, comparison of the top mRNAs that were more abundant in both young individuals and those living above poverty showed that out of the top 20 hits 6 overlapped including *IL1B, EIF1AY, CCL3L3, FAM96A, HLA-DRB1,* and *ATRAID* (Figure 4C; Tables 4, 5). Out of the top 20 mRNAs that were less abundant with age and poverty status *ETS1, C20orf152, NANOG,* and *SLC18A3* were all overlapping (Figure 4C; Tables 4, 5).

**Table 4:** Top changes in mRNA abundance with age.

**Increased in Young**	**Decreased in Young**

**Gene Symbol**	**Fold**	***P* value**	**Seqname**	**Gene Symbol**	**Fold**	***P* value**	**Seqname**
***IL1B***	13.23	0.027	NM_000576	***CSMD2***	30.48	0.002	ENST00000241312
***EIF1AY***	12.58	0.027	ENST00000361365	***PLCD3***	25.30	0.000	NM_133373
***CCL3L3***	11.02	0.009	NM_001001437	***KRTAP9-9***	24.11	0.003	ENST00000394008
***FAM216A***	10.80	0.002	NM_013300	***KRTAP9-4***	19.41	0.002	NM_033191
***FGFBP2***	10.32	0.002	NM_031950	***C20orf78***	17.73	0.003	NM_001242671
***FAM96A***	9.42	0.003	NM_001014812	***C4orf26***	14.59	0.000	NM_178497
***HIGD2A***	9.33	0.003	NM_138820	***LRR1***	14.36	0.000	NM_203467
***TPI1***	8.50	0.000	NM_000365	***PARP16***	14.22	0.001	NM_017851
***CLEC4D***	8.46	0.004	NM_080387	***ZNF442***	14.17	0.002	NM_030824
***SPTLC2***	7.95	0.001	NM_004863	***CABLES1***	13.31	0.005	NM_138375
***HLA-DRB1***	7.93	0.014	NM_002124	***IFIT3***	11.77	0.002	NM_001549
***TBXAS1***	7.89	0.018	NM_001166254	***ETS1***	11.35	0.018	NM_005238
***COMMD3***	7.84	0.000	NM_012071	***CR1***	11.21	0.004	NM_000651
***TXN***	7.63	0.000	NM_003329	***C20orf152***	10.51	0.002	ENST00000349339
***ATRAID***	7.63	0.020	NM_016085	***NANOG***	9.89	0.005	NM_024865
***XRCC5***	7.45	0.016	NM_021141	***SLC18A3***	9.55	0.003	NM_003055
***AP1M1***	7.41	0.000	ENST00000291439	***ADCY6***	9.17	0.004	NM_015270
***PDXK***	7.21	0.005	NM_003681	***HOXC4***	8.89	0.000	ENST00000430889
***ERH***	7.15	0.000	NM_004450	***KRTAP2-4***	8.60	0.002	NM_033184
***GSTO1***	7.03	0.004	NM_001191003	***DKK1***	8.56	0.002	NM_012242

Top mRNAs differentially expressed with age and *P*<0.05, Fold change >2 and FDR <0.1.

**Table 5:** Top changes in mRNA abundance with poverty.

**Increased in Above Poverty**				**Decreased in Above Poverty**			
**Gene Symbol**	**Fold**	***P* value**	**Seqname**	**Gene Symbol**	**Fold**	***P* value**	**Seqname**
***EIF1AY***	16.03	0.014	ENST00000361365	***ETS1***	11.59	0.018	NM_005238
***IL1B***	14.94	0.020	NM_000576	***AMMECR1L***	8.72	0.006	NM_031445
***CST7***	11.06	0.004	ENST00000480798	***CDC14B***	8.02	0.011	NM_003671
***HLA-DQB1***	10.06	0.003	NM_002123	***DDR1***	7.64	0.005	NM_001954
***ATRAID***	9.66	0.008	NM_016085	***NANOG***	7.34	0.021	NM_024865
***TREM1***	9.46	0.005	NM_018643	***SOX12***	6.43	0.016	NM_006943
***FLJ44635***	9.26	0.005	NM_207422	***MBD3L1***	6.39	0.004	ENST00000305625
***HLA-DRB1***	9.05	0.008	NM_002124	***GTPBP3***	5.66	0.022	NM_133644
***SAT1***	8.75	0.004	NM_002970	***SLC18A3***	5.63	0.035	NM_003055
***NFKBIA***	8.73	0.005	NM_020529	***ZDHHC8***	5.53	0.026	NM_013373
***CCL3L3***	8.67	0.023	NM_001001437	***C20orf152***	5.50	0.043	ENST00000349339
***CLEC4E***	8.66	0.003	NM_014358	***DCN***	5.47	0.003	NM_133504
***CUTC***	8.64	0.004	NM_015960	***SYT4***	5.31	0.029	NM_020783
***OAZ1***	8.62	0.004	NM_004152	***PTF1A***	5.28	0.041	NM_178161
***RPS8***	8.44	0.001	NM_001012	***FAM153B***	5.27	0.006	ENST00000253490
***JTB***	8.31	0.004	NM_006694	***MAGEA6***	5.24	0.001	NM_175868
***ISCU***	8.16	0.002	NM_213595	***CPSF1***	5.23	0.010	NM_013291
***NPM1***	8.07	0.007	NM_199185	***DCTN1***	5.14	0.015	NM_001135041
***FAM96A***	8.01	0.003	NM_032231	***CT45A1***	5.07	0.005	NM_001017417
***SMYD2***	7.97	0.002	NM_020197	***CPN1***	4.98	0.005	NM_001308

Top mRNAs differentially expressed with poverty and *P*<0.05, Fold change >2 and FDR <0.1.

Differences in mRNA abundance were also examined in AA males. There were very few mRNAs showing significant changes in abundance with age in AA males (Supplementary Figure 1). More mRNAs were significantly different comparing individuals above and below poverty (Supplementary Figure 1).

### Age and poverty pathway analysis

**Figure 5:**
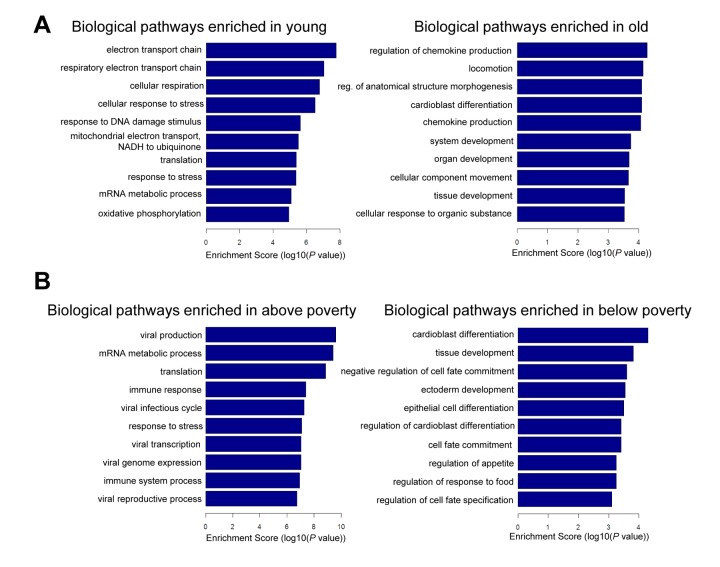
**Pathway analysis for age and poverty.** Differentially expressed mRNAs with age and poverty were used for gene ontology (GO) analysis using categories derived from Gene Ontology. Top biological pathways are shown for age (**A**) and poverty (**B**).

To gain additional information about the biological pathways that may be regulated by the mRNAs changing in abundance with poverty and age, we performed gene ontology (GO) analysis. In younger individuals, we found enrichment of pathways associated with mitochondrial function and response to DNA damage and stress (Figure 5A). In older individuals, pathways related to chemokine production and development were enriched. In the context of poverty, pathways related to development and differentiation were enriched in individuals living below poverty (Figure 5B). We found that the top pathways enriched among those living above poverty were those implicated in the response to stress, immune stimuli, and viral infection (Figure 5B). This finding is not unexpected because mRNAs increased in abundance in response to social adversity, are part of the inflammatory response cascade, and mRNAs decreased in abundance with social adversity are noted to be related to the innate interferon antiviral response as well as synthesis of antibodies [27].

## DISCUSSION

Here, we have examined differences in expressed lncRNAs and mRNAs with age and poverty status in both white and AA males. In our initial genome-wide profiling, we found that white males had larger significant differences in expression levels of lncRNAs and mRNAs with poverty and age, as compared to AA males, where these changes were far more modest. This indicates that the influence of age and poverty on gene expression patterns differs between racial groups. It is not clear what drives these differences, but they may correlate with the wide gap in health status and health outcomes between AAs and whites particularly between AA men and white men. AA males living below poverty in the HANDLS cohort, and in other studies, are particularly vulnerable to early mortality compared to their white counterparts and even when compared to AA men who live above the poverty line despite adjustments for various lifestyle cofactors [21,22]. Given this disparity, it is important to examine whether and how gene expression or other genomic factors may act as a biologic transduction pathway for the social determinants of health.

We found altered expression levels in a number of lncRNAs with age and poverty in white males. Although lncRNAs have been studied in the context of aging, in general most studies have focused on a specific lncRNA or on those that relate to various hallmarks of aging. Here, we have performed a comprehensive study of lncRNAs that change in abundance with human age in men whose life expectancy at birth and life span is shorter than women. We also compared these lncRNAs to a growing list of age-associated lncRNAs from the literature. Several of these overlapping lncRNAs were further validated in an expanded cohort of individuals; among these, *GAS5, H19, TERC* and *MEG3* levels were all significantly changed with age (Figure 3B). *GAS5 and MEG3* levels were significantly different with poverty. These data further confirm that these are true age-associated lncRNAs, particularly since these lncRNAs were identified as being aging-associated in other tissues or model systems. Several of these lncRNAs regulate aging processes including telomere function (*TERC*), autophagy (*MEG3*), protein trafficking (*GAS5*), and epigenetic change and proliferation (*H19*) [13]. Investigators have found that many of these lncRNAs participate in regulating age-related disease [15].

In addition to confirming these age-associated lncRNAs, we also identified many novel age- and poverty-associated lncRNAs (Tables 2, 3). The lncRNAs displaying the largest increases and decreases in abundance were further validated in our expanded cohort; *CTD-3247F14.2 (*LNCipedia: *lnc-EGR3-1)* levels were significantly changed with age, while *AK022914 (DUXAP9),* *KB-1047C11.2 (*LNCipedia: *C8orf37-AS1:1),* and *XLOC_003262* (LNCipedia: *lnc-KY-1*) levels were significantly changed with poverty. These data shed new light on various lncRNAs that may be important biological transducers of stressors related to poverty and age, as well as to the loss of homeostasis that may result from both. Future work lies in determining whether the expression of these lncRNAs is a consequence or contributor to the aging process. Interestingly, we found that the levels of one lncRNA, *RNF157-AS1*, were altered differentially with age in AAs and in whites. It will be interesting in the future to further investigate how this lncRNA expression is altered with age and race.

In summary, we have identified lncRNAs that are altered in abundance with both poverty and age in whites and AAs. As our knowledge of the functional roles of lncRNAs expands, it will be important to determine how these ncRNAs contribute to the aging process and to the biological transduction of social adversity and social determinants that result in health disparities.

## MATERIALS AND METHODS

### Clinical participants

Participants were chosen from the Healthy Aging in Neighborhoods of Diversity across the Life Span (HANDLS) study of the National Institute on Aging Intramural Research Program, National Institutes of Health (NIH). The study has been approved by the Institutional Review Board of the National Institute of Environmental Health Sciences, NIH. All participants provided written informed consent. HANDLS is a Baltimore, Maryland based longitudinal epidemiologic study focused on examining the interaction of race and socioeconomic status on aging and age-related health disparities [37]. A sub-cohort of young (~30 yrs) and old (~64 yrs) white or African American (AA) males above or below poverty were chosen for the microarray (n=8/group) (Table 1). An expanded cohort of white males above and below poverty were chosen for RT-qPCR validation (n=20/group; Table 1). Below poverty was designated if the self-reported household income was below 125% of the 2004 Health and Human Services Poverty Guidelines at baseline recruitment.

### PBMCs

Fasting blood samples are obtained from participants in the morning and collected in 8-ml Vacutainer® heparinized vials (BD, Franklin Lakes, NJ). Peripheral blood mononuclear cells were isolated within 3 hours of phlebotomy as detailed previously [4]. After isolation, PBMCs are aliquoted and stored at -80^o^C.

### RNA isolation and microarray

Total RNA from PBMCs was isolated using TRIzol™ according to manufacturer’s instructions. RNA quality and quantity were analyzed using a NanoDrop ND-1000 spectrophotometer and standard denaturing agarose gel electrophoresis.

lncRNA and mRNA microarrays were performed by Arraystar Inc. The Arraystar Human LncRNA Microarray V3.0 contains lncRNAs identified through public transcriptome databases (Refseq, UCSC knowngenes, Gencode, etc), as well as from the literature. In brief, sample preparation and microarray hybridization were performed according to the Agilent One-Color Microarray-Based Gene Expression Analysis protocol (Agilent Technology) with minor modifications. mRNA was purified from total RNA after removal of rRNA (mRNA-ONLY™ Eukaryotic mRNA Isolation Kit, Epicentre). Subsequently, each sample was amplified and transcribed into fluorescent cRNA along the entire length of the transcripts without 3’ bias utilizing a random priming method (Arraystar Flash RNA Labeling Kit, Arraystar). The labeled cRNAs were hybridized onto the Human LncRNA Array v3.0 (8 x 60K, Arraystar). After washing and fixing of the slides, the arrays were scanned by the Agilent Scanner G2505C.

Acquired array images were analyzed using the Agilent Feature Extraction software (version 11.0.1.1). Quantile normalization and subsequent data processing were performed using the GeneSpring GX v12.0 software package (Agilent Technologies). After quantile normalization of the raw data, ComBat was used to adjust batch effects. After normalization of the raw data, lncRNAs and mRNAs that at least 8 out of 16 samples have flags in present or marginal were chosen for further data analysis. Differentially expressed lncRNAs and mRNAs with statistical significance with Fold Change >= 2.0, *P* value <= 0.05 were identified through Volcano Plot filtering between two groups. All significantly changed lncRNAs are listed in Supplementary Table 1, Supplementary Table 2, Supplementary Table 3, Supplementary Table 4 and mRNAs in Supplementary Table 6, Supplementary Table 7, Supplementary Table 8, Supplementary Table 9. Differentially expressed mRNAs were used for gene ontology (GO) analysis using categories derived from Gene Ontology (http://www.geneontology.org). Biological pathways are shown. Fisher’s exact test was used to calculate significance and *P* value denotes the significance of GO term enrichment in the differentially expressed genes. Microarray data can be accessed at GEO (Accession Number: GSE123500).

### RT-qPCR

Cryopreserved PBMCs were thawed quickly and washed with PBS. Total RNA was isolated using TRIzol™ according to manufacturer’s instructions with an inclusion of a DNase treatment step. RNA quality and quantity were analyzed using a NanoDrop 2000c. RNA was reverse transcribed using random hexamers and reverse transcriptase (Invitrogen). RT-qPCR was performed using SYBR green master mix and lncRNA specific primers. LncRNA primer sequences were designed using NCBI reference sequences or sequences from LNCipedia (www.lncipedia.org). Primer sequences and detailed information are listed in Supplementary Table 10. In some cases, the LNCipedia nomenclature differs from the nomenclature used here and these alternative names are also listed in the text and in Supplementary Table 10. *NEAT1* and *MEG3* primers were described previously [38,39]. *GAS5* has 29 different transcripts and primers were designed using sequences from microarray. Similar data was observed for transcript variants *GAS5_019, GAS5_24* and *GAS5_026* and data only from *GAS5_019* is shown. For other lncRNAs with multiple variants, primers were designed against exons that overlapping between the variants. Reactions were run on an Applied Biosystems 7500 Real-Time PCR System using default settings. lncRNA expression was normalized to an average of *HPRT* and *UBC*. Previously we have found that these mRNAs are least variable for aging studies in PBMCs [40]. lncRNA levels were examined for Gaussian distribution by measuring kurtosis and skewness and outliers for each lncRNA were excluded from the analysis using Grubb’s test with an alpha of 0.05.

## Supplementary Material

Supplementary Figure

Supplementary Table 1

Supplementary Table 2

Supplementary Table 3

Supplementary Table 4

Supplementary Table 5

Supplementary Table 6

Supplementary Table 7

Supplementary Table 8

Supplementary Table 9

Supplementary Table 10

## References

[1] López-Otín C, Blasco MA, Partridge L, Serrano M, Kroemer G. The hallmarks of aging. Cell. 2013; 153:1194–217. 10.1016/j.cell.2013.05.039 23746838 PMC3836174

[2] Abdelmohsen K, Gorospe M. Noncoding RNA control of cellular senescence. Wiley Interdiscip Rev RNA. 2015; 6:615–29. 10.1002/wrna.1297 26331977 PMC4562317

[3] Jung HJ, Suh Y. MicroRNA in Aging: From Discovery to Biology. Curr Genomics. 2012; 13:548–57. 10.2174/138920212803251436 23633914 PMC3468887

[4] Noren Hooten N, Abdelmohsen K, Gorospe M, Ejiogu N, Zonderman AB, Evans MK. microRNA expression patterns reveal differential expression of target genes with age. PLoS One. 2010; 5:e10724. 10.1371/journal.pone.0010724 20505758 PMC2873959

[5] Noren Hooten N, Fitzpatrick M, Wood WH 3rd, De S, Ejiogu N, Zhang Y, Mattison JA, Becker KG, Zonderman AB, Evans MK. Age-related changes in microRNA levels in serum. Aging (Albany NY). 2013; 5:725–40. 10.18632/aging.100603 24088671 PMC3838776

[6] Smith-Vikos T, Liu Z, Parsons C, Gorospe M, Ferrucci L, Gill TM, Slack FJ. A serum miRNA profile of human longevity: findings from the Baltimore Longitudinal Study of Aging (BLSA). Aging (Albany NY). 2016; 8:2971–87. 10.18632/aging.101106 27824314 PMC5191881

[7] Noren Hooten N, Martin-Montalvo A, Dluzen DF, Zhang Y, Bernier M, Zonderman AB, Becker KG, Gorospe M, de Cabo R, Evans MK. Metformin-mediated increase in DICER1 regulates microRNA expression and cellular senescence. Aging Cell. 2016; 15:572–81. 10.1111/acel.12469 26990999 PMC4854919

[8] Smith-Vikos T, de Lencastre A, Inukai S, Shlomchik M, Holtrup B, Slack FJ. MicroRNAs mediate dietary-restriction-induced longevity through PHA-4/FOXA and SKN-1/Nrf transcription factors. Curr Biol. 2014; 24:2238–46. 10.1016/j.cub.2014.08.013 25242029 PMC4208828

[9] Boehm M, Slack F. A developmental timing microRNA and its target regulate life span in C. elegans. Science. 2005; 310:1954–57. 10.1126/science.1115596 16373574

[10] Smith-Vikos T, Slack FJ. MicroRNAs and their roles in aging. J Cell Sci. 2012; 125:7–17. 10.1242/jcs.099200 22294612 PMC3269020

[11] Kopp F, Mendell JT. Functional Classification and Experimental Dissection of Long Noncoding RNAs. Cell. 2018; 172:393–407. 10.1016/j.cell.2018.01.011 29373828 PMC5978744

[12] Noh JH, Kim KM, McClusky WG, Abdelmohsen K, Gorospe M. Cytoplasmic functions of long noncoding RNAs. Wiley Interdiscip Rev RNA. 2018; 9:e1471. 10.1002/wrna.1471 29516680 PMC5963534

[13] Grammatikakis I, Panda AC, Abdelmohsen K, Gorospe M. Long noncoding RNAs(lncRNAs) and the molecular hallmarks of aging. Aging (Albany NY). 2014; 6:992–1009. 10.18632/aging.100710 25543668 PMC4298369

[14] Kim C, Kang D, Lee EK, Lee JS. Long Noncoding RNAs and RNA-Binding Proteins in Oxidative Stress, Cellular Senescence, and Age-Related Diseases. Oxid Med Cell Longev. 2017; 2017:2062384. 10.1155/2017/2062384 28811863 PMC5547732

[15] Kim J, Kim KM, Noh JH, Yoon JH, Abdelmohsen K, Gorospe M. Long noncoding RNAs in diseases of aging. Biochim Biophys Acta. 2016; 1859:209–21. 10.1016/j.bbagrm.2015.06.013 26141605 PMC4698248

[16] Stegeman R, Weake VM. Transcriptional Signatures of Aging. J Mol Biol. 2017; 429:2427–37. 10.1016/j.jmb.2017.06.019 28684248 PMC5662117

[17] Crews DC, Charles RF, Evans MK, Zonderman AB, Powe NR. Poverty, race, and CKD in a racially and socioeconomically diverse urban population. Am J Kidney Dis. 2010; 55:992–1000. 10.1053/j.ajkd.2009.12.032 20207457 PMC2876201

[18] Odutayo A, Gill P, Shepherd S, Akingbade A, Hopewell S, Tennankore K, Hunn BH, Emdin CA. Income Disparities in Absolute Cardiovascular Risk and Cardiovascular Risk Factors in the United States, 1999-2014. JAMA Cardiol. 2017; 2:782–90. 10.1001/jamacardio.2017.1658 28593301 PMC5710615

[19] Stringhini S, Carmeli C, Jokela M, Avendaño M, Muennig P, Guida F, Ricceri F, d’Errico A, Barros H, Bochud M, Chadeau-Hyam M, Clavel-Chapelon F, Costa G, et al, and LIFEPATH consortium. Socioeconomic status and the 25 × 25 risk factors as determinants of premature mortality: a multicohort study and meta-analysis of 1·7 million men and women. Lancet. 2017; 389:1229–37. 10.1016/S0140-6736(16)32380-7 28159391 PMC5368415

[20] Geronimus AT. The weathering hypothesis and the health of African-American women and infants: evidence and speculations. Ethn Dis. 1992; 2:207–21. 1467758

[21] Sloan FA, Ayyagari P, Salm M, Grossman D. The longevity gap between Black and White men in the United States at the beginning and end of the 20th century. Am J Public Health. 2010; 100:357–63. 10.2105/AJPH.2008.158188 20019309 PMC2804648

[22] Zonderman AB, Mode NA, Ejiogu N, Evans MK. Race and poverty status as a risk for overall mortality in community-dwelling middle-aged adults. JAMA Intern Med. 2016; 176:1394–95. 10.1001/jamainternmed.2016.3649 27428269 PMC5831185

[23] Kochanek K, Murphy S, Xu J, Arias E. Mortality in the United States, 2016. NCHS Data Brief, no 293. Hyattsville, MD: National Center for Health Statistics. 2017. 29319473

[24] Bilal U, Diez-Roux AV. Troubling Trends in Health Disparities. N Engl J Med. 2018; 378:1557–58. 10.1056/NEJMc1800328 29669223

[25] Gaye A, Gibbons GH, Barry C, Quarells R, Davis SK. Influence of socioeconomic status on the whole blood transcriptome in African Americans. PLoS One. 2017; 12:e0187290. 10.1371/journal.pone.0187290 29206834 PMC5716587

[26] Powell ND, Sloan EK, Bailey MT, Arevalo JM, Miller GE, Chen E, Kobor MS, Reader BF, Sheridan JF, Cole SW. Social stress up-regulates inflammatory gene expression in the leukocyte transcriptome via β-adrenergic induction of myelopoiesis. Proc Natl Acad Sci USA. 2013; 110:16574–79. 10.1073/pnas.1310655110 24062448 PMC3799381

[27] Cole SW. Human social genomics. PLoS Genet. 2014; 10:e1004601. 10.1371/journal.pgen.1004601 25166010 PMC4148225

[28] Slavich GM, Cole SW. The Emerging Field of Human Social Genomics. Clin Psychol Sci. 2013; 1:331–48. 10.1177/2167702613478594 23853742 PMC3707393

[29] Hero JO, Zaslavsky AM, Blendon RJ. The United States Leads Other Nations In Differences By Income In Perceptions Of Health And Health Care. Health Aff (Millwood). 2017; 36:1032–40. 10.1377/hlthaff.2017.0006 28583961

[30] Seeman TE, Crimmins E. Social environment effects on health and aging: integrating epidemiologic and demographic approaches and perspectives. Ann N Y Acad Sci. 2001; 954:88–117. 10.1111/j.1749-6632.2001.tb02749.x 11797869

[31] Abdelmohsen K, Panda A, Kang MJ, Xu J, Selimyan R, Yoon JH, Martindale JL, De S, Wood WH 3rd, Becker KG, Gorospe M. Senescence-associated lncRNAs: senescence-associated long noncoding RNAs. Aging Cell. 2013; 12:890–900. 10.1111/acel.12115 23758631 PMC3773026

[32] Peffers MJ, Fang Y, Cheung K, Wei TK, Clegg PD, Birch HL. Transcriptome analysis of ageing in uninjured human Achilles tendon. Arthritis Res Ther. 2015; 17:33. 10.1186/s13075-015-0544-2 25888722 PMC4355574

[33] Puvvula PK, Desetty RD, Pineau P, Marchio A, Moon A, Dejean A, Bischof O. Long noncoding RNA PANDA and scaffold-attachment-factor SAFA control senescence entry and exit. Nat Commun. 2014; 5:5323. 10.1038/ncomms6323 25406515 PMC4263151

[34] Bianchessi V, Badi I, Bertolotti M, Nigro P, D’Alessandra Y, Capogrossi MC, Zanobini M, Pompilio G, Raucci A, Lauri A. The mitochondrial lncRNA ASncmtRNA-2 is induced in aging and replicative senescence in Endothelial Cells. J Mol Cell Cardiol. 2015; 81:62–70. 10.1016/j.yjmcc.2015.01.012 25640160

[35] Lai KM, Gong G, Atanasio A, Rojas J, Quispe J, Posca J, White D, Huang M, Fedorova D, Grant C, Miloscio L, Droguett G, Poueymirou WT, et al. Diverse Phenotypes and Specific Transcription Patterns in Twenty Mouse Lines with Ablated LincRNAs. PLoS One. 2015; 10:e0125522. 10.1371/journal.pone.0125522 25909911 PMC4409293

[36] Mourtada-Maarabouni M, Pickard MR, Hedge VL, Farzaneh F, Williams GT. GAS5, a non-protein-coding RNA, controls apoptosis and is downregulated in breast cancer. Oncogene. 2009; 28:195–208. 10.1038/onc.2008.373 18836484

[37] Evans MK, Lepkowski JM, Powe NR, LaVeist T, Kuczmarski MF, Zonderman AB. Healthy aging in neighborhoods of diversity across the life span (HANDLS): overcoming barriers to implementing a longitudinal, epidemiologic, urban study of health, race, and socioeconomic status. Ethn Dis. 2010; 20:267–75. 20828101 PMC3040595

[38] Mondal T, Subhash S, Vaid R, Enroth S, Uday S, Reinius B, Mitra S, Mohammed A, James AR, Hoberg E, Moustakas A, Gyllensten U, Jones SJ, et al. MEG3 long noncoding RNA regulates the TGF-β pathway genes through formation of RNA-DNA triplex structures. Nat Commun. 2015; 6:7743. 10.1038/ncomms8743 26205790 PMC4525211

[39] Yoon JH, De S, Srikantan S, Abdelmohsen K, Grammatikakis I, Kim J, Kim KM, Noh JH, White EJ, Martindale JL, Yang X, Kang MJ, Wood WH 3rd, et al. PAR-CLIP analysis uncovers AUF1 impact on target RNA fate and genome integrity. Nat Commun. 2014; 5:5248. 10.1038/ncomms6248 25366541 PMC4291169

[40] Cabili MN, Trapnell C, Goff L, Koziol M, Tazon-Vega B, Regev A, Rinn JL. Integrative annotation of human large intergenic noncoding RNAs reveals global properties and specific subclasses. Genes Dev. 2011; 25:1915–27. 10.1101/gad.17446611 21890647 PMC3185964

