# Supplementary material for: Age and poverty status alter the coding and noncoding transcriptome
Source: Aging (Albany NY). 2019 Feb 17;11(4):1189–203. doi: 10.18632/aging.101823 (PMC6402526; doi:10.18632/aging.101823)
Supplement: Supplementary Figure [file aging-11-101823-s001.pdf]

SUPPLEMENTARY FIGURE

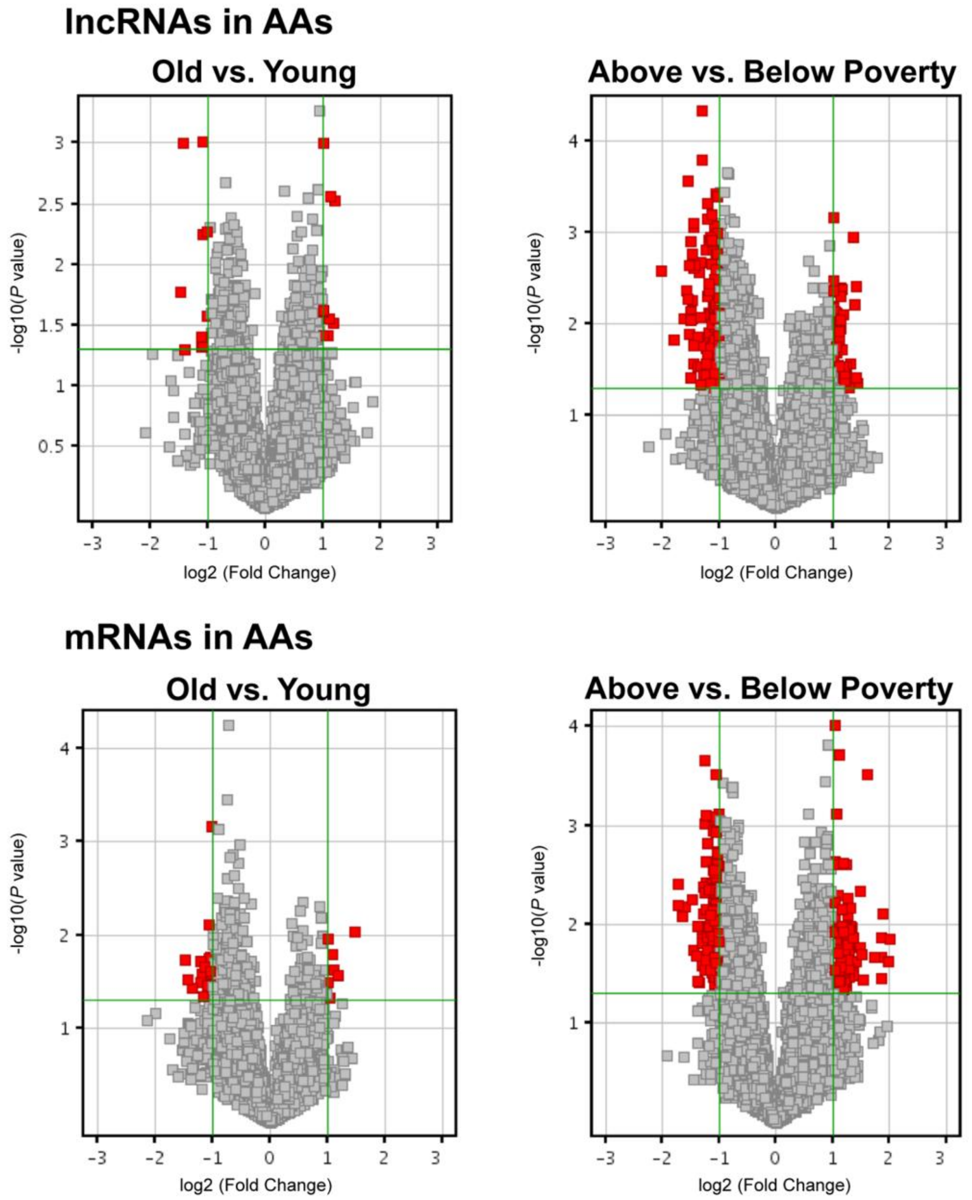

Supplementary Figure 1. lncRNA and mRNA level changes with age and poverty in African American males. lncRNA and mRNA expression from African American (AA) young and old males living above or below poverty was assessed using microarray (Table 1A for demographic details). Volcano plots show log2 fold change and P value for each lncRNA and mRNA. Red indicates lncRNAs and mRNAs that were >2 fold change and *P* value for each lncRNA and mRNA. Red indicateslncRNAs and mRNAs that were >2 fold change and *P*<0.05. Comparisons between groups are indicated.
